# Poincaré plot analysis of cerebral blood flow signals: Feature extraction and classification methods for apnea detection

**DOI:** 10.1371/journal.pone.0208642

**Published:** 2018-12-07

**Authors:** Carmen González, Erik W. Jensen, Pedro L. Gambús, Montserrat Vallverdú

**Affiliations:** 1 Biomedical Engineering Research Centre, Universitat Politècnica de Catalunya, CIBER of Bioengineering, Biomaterials and Nanomedicine (CIBER-BBN), Barcelona, Spain; 2 Quantium Medical, Research and Development Department, Mataró, Spain; 3 Hospital Clínic de Barcelona, Department of Anaesthesiology, Barcelona, Spain; Universidad de Granada, SPAIN

## Abstract

**Objective:**

Rheoencephalography is a simple and inexpensive technique for cerebral blood flow assessment, however, it is not used in clinical practice since its correlation to clinical conditions has not yet been extensively proved. The present study investigates the ability of Poincaré Plot descriptors from rheoencephalography signals to detect apneas in volunteers.

**Methods:**

A group of 16 subjects participated in the study. Rheoencephalography data from baseline and apnea periods were recorded and Poincaré Plot descriptors were extracted from the reconstructed attractors with different time lags (τ). Among the set of extracted features, those presenting significant differences between baseline and apnea recordings were used as inputs to four different classifiers to optimize the apnea detection.

**Results:**

Three features showed significant differences between apnea and baseline signals: the Poincaré Plot ratio (SDratio), its correlation (R) and the Complex Correlation Measure (CCM). Those differences were optimized for time lags smaller than those recommended in previous works for other biomedical signals, all of them being lower than the threshold established by the position of the inflection point in the CCM curves. The classifier showing the best performance was the classification tree, with 81% accuracy and an area under the curve of the receiver operating characteristic of 0.927. This performance was obtained using a single input parameter, either SDratio or R.

**Conclusions:**

Poincaré Plot features extracted from the attractors of rheoencephalographic signals were able to track cerebral blood flow changes provoked by breath holding. Even though further validation with independent datasets is needed, those results suggest that nonlinear analysis of rheoencephalography might be a useful approach to assess the correlation of cerebral impedance with clinical changes.

## Introduction

Continuous monitoring of cerebral blood flow (CBF) is critical as it reflects the amount of blood provided to the brain and therefore the amount of oxygen that reaches this organ. An insufficient or excessive perfusion can have deleterious effects in patient’s health and for this reason continuous CBF monitoring could increase patient safety.

Zauner and Muizelaar [1] described the characteristics of the ideal CBF monitor stating that it should provide quantitative information, high spatial resolution, continuous measurements, have no influence on normal brain function, represent none or minimal risk for the patient, be cost-effective and suitable for clinical settings. Several monitors are currently available in clinical practice for CBF monitoring but they present limitations since some of the most accurate ones are either invasive or expensive.

Among the existing methods for CBF monitoring, rheoencephalography (REG) is one of the most cost-effective solutions but it is not used in clinical practice because reported results have not been able to provide clear correlations to clinical scenarios [2]. REG is an explorative method of cerebral circulation based on the measurement of electrical impedance through the scalp, which allows a continuous observation of the blood flow in different cerebral regions. The principle of this method is based upon the fact that blood is a good electrical conductor therefore any increase in blood volume will lead to a reduction of the brain electrical resistance and this will be reflected in a decrease of REG pulse amplitude given a constant current.

REG signals have traditionally been analyzed by assessing the geometrical properties of the blood pulse waves in the time domain, such as the duration of the anacrotic phase of the pulse, the maximum and minimum amplitudes, the slope and the area under the curve [3,4]. The main limitation of REG data processing is the existence of artifacts, such as respiration or movements, differences in tissue conductivity, and the lack of absolute measurements of CBF, due to the fact that REG only provides information on relative changes in blood flow. Despite these facts, since it is a non-invasive and very low cost technique, it is still attractive for researchers [2]. REG was extensively used during the 1960s and 1970s but its popularity decayed because the provided results were non-conclusive from a clinical viewpoint. Nowadays, with more precise electronics, software capabilities and newer methods for CBF assessment such as image based techniques, which are very expensive but can be used as a reference for validation purposes, investigations on REG are beginning to show signs of uptake once more. [5].

One strategy used to evaluate CBF measurements consists of continuous assessment of blood flow during the execution of respiratory challenges known to modify the CBF. For example, episodes of apnea or breath holding reduce the amount of oxygen in blood and therefore partial CO2 pressure increases provoking increases in CBF. Kastrup et al. [6] quantified the effect of an apnea procedure in regional CBF measured with magnetic resonance imaging and, on average, found a regional CBF increase of 47–87%, dependent on apnea duration. Increasing inhaled CO2 [7] is an alternative method that can be used to provoke changes in CBF that has also been widely used though its implementation is far more complex since it requires controlled CO2 inhalation.

The challenges described would cause changes in cerebral perfusion however CBF is affected by additional factors. Biological signals are known to be controlled by central nervous system oscillators that make them complex, causing some irregular patterns [8]. Nonetheless, some underlying well determined behavior exists. These signals could probably be better characterized by dynamic nonlinear analysis instead of using standard linear time series signal processing techniques.

One nonlinear technique used to study beat to beat intervals is the method of delayed coordinates for state-space analysis, the so-called Poincaré plot. Dimitriev et al. [9] analyzed by means of nonlinear dynamics based on Poincaré plots how the state of anxiety affected heart rate variability. Voss et al. [10] have previously published on the effects of age and gender in short-term heart rate variability analyzed with Poincaré plots among other features and Ebrahimzadeh [11] explored the prediction of sudden cardiac death based on complexity analysis. Other biological signals have been studied by means of Poincaré plots. Hayashi [12] related the delayed coordinates map to changes provoked by anesthesia in the electroencephalograph (EEG). Xiong et al. [13] explored the ability of Poincaré plots from electromyogram (EMG) to reflect facial paralysis and Son et al. [14] studied regularity in respiratory signals using this same technique.

Hoshi et al. [15] used standard descriptors of Poincaré plot analysis to distinguish between healthy subjects and patients suffering coronary disease, concluding that the SD1/SD2 index provided useful information for that purpose. Even though some features extracted from Poincaré plots are known to be highly correlated to linear time domain information [16], some others reflect nonlinear behaviors, complementing the diagnosis capabilities of heart rate variability signals, such as the SD1/SD2 parameter or the Complex Correlation Measure [17].

Poincaré Plot Analysis is typically applied with a time lag of 1 sample, therefore plotting the original signal versus its 1 sample delayed version. Several publications have explored the possibility of using different time lags to build the Poincaré plot. Since consecutive samples are highly correlated, when a lag of 1 sample is used, data are concentrated on the identity line. Increasing time lags would spread the data points over the Poincaré plot, because there is less correlation between lagged samples [18,19].

Brennan et al. [16] discussed the effects of lagging the Poincaré plots, showing that those lagged plots characterize the autocovariance function, yet there is no consensus on which lags should be used [20]. Lerma et al. [21] determined that a lag of 4 heart beats would optimize the detection of changes in heart rate variability due to hemodialysis in chronic renal failure patients, while Thakre and Smith [19] stated that a heartbeat can only influence up to the next 6 to 8 beats and therefore higher lags would not be suitable for those applications. Therefore, lags lower than 10 are typically used for RR intervals [20].

Contreras et al. [22] used lagged Poincaré plots and assessed the correlation between the spectral features (HF and LF) and the SD1 parameter, concluding that the value of those correlations was different healthy and pathological heart rate variability signals. Lagged plots were also used by Goshvarpour [18] to analyze heart rate during mediation, detecting an increase in the SD1 parameter for increasing lags up to 6 beats which reflects the transition between cigar-shaped plots for the smallest lag to a cloud of points with the largest ones.

The preesent paper focuses on the analysis of the dynamics of REG signals through lagged Poincaré plots aiming at understanding underlying nonlinear behavior and identifying how those dynamics can assess physiological changes affecting the system. Since CBF is modulated by several physiological conditions, applying nonlinear analysis could be a very promising tool for improving clinical information extracted from REG. The analysis herein proposed is therefore based on the method of delayed coordinates for state-space analysis since, to the extent of the knowledge of the authors, it has not been used for REG data processing. A simple respiratory challenge, apnea, was used to explore REG capability to reflect CBF changes and to analyze nonlinear dynamics in REG signals. Results obtained from this analysis were compared to the ones provided by standard analysis using the geometrical properties of the pulses. Finally, four different classifiers were applied to the Poincaré plots extracted features to explore the possibility of predicting apneas using information on the nonlinear dynamics of REG signals.

## Database

**Fig 1:**
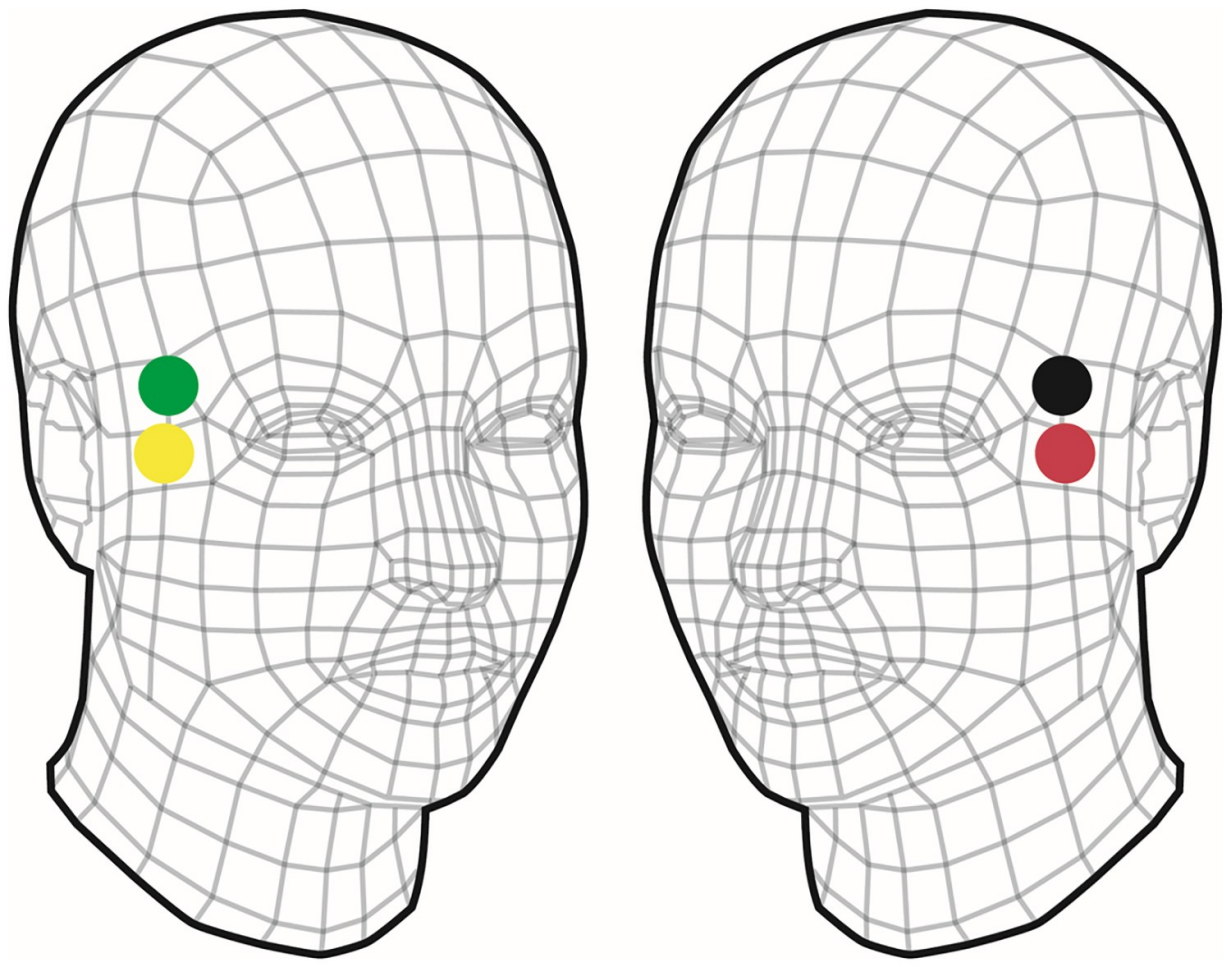
Electrodes placement for cerebral bioimpedance recordings to measure cerebral blood flow.

Participants were 16 healthy volunteers aged 25.4 ±3.6 years, 59.6 ± 6.8 kg weight and 166.9 ±8.3 cm height, including 8 males and 8 females. Four pre-gelled standard Ag/AgCl ECG electrodes were placed on the subject temples as shown in Fig 1, two electrodes (red and yellow), one on each temple, were used to send a constant 50kHz current through the scalp while the other pair of electrodes (green and black) were responsible for measuring the output voltage at a sampling rate of 250Hz. Since current is constant, this voltage reflects the effects of impedance changes in the brain caused by variation of blood flows. The qCO monitor (Quantium Medical, Barcelona, Spain) was used to monitor the cerebral bioimpedance signal (REG) for cerebral blood flow (CBF) estimation.

The subjects were monitored in supine position for 8 minutes repeating twice the sequence of 3 minutes at rest followed by 1 minute of breath holding. Subjects were asked to avoid talking, movements and blinking, since those would provoke artifacts. Apneas were planned to stand for 1 minute however volunteers were instructed to stop earlier if needed and raise their hand to communicate to the investigator that the apnea period was over. All subjects were informed about the study and gave their written informed consent prior to participation. This observational study was conducted under approval of the Institutional Review Board and Ethics Committee of Hospital CLINIC de Barcelona (2013/8356) and adhered to the Declaration of Helsinki.

## Methodology

### Signal preprocessing

**Fig 2:**
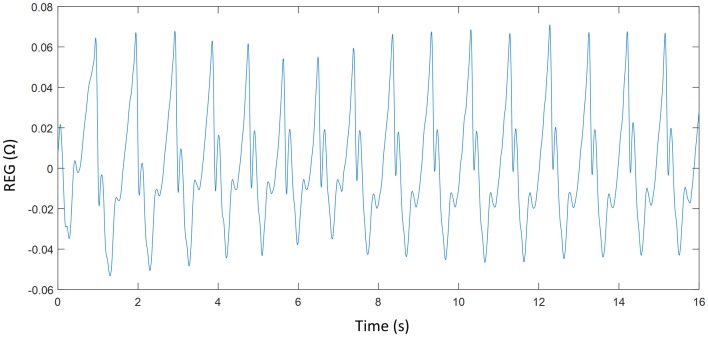
Waveform of a filtered REG signal of an apnea segment.

The collected REG signals were pre-processed. REG recordings were high-pass filtered using 4th-order Chebyshev type II, with 0.1 Hz stop band frequency to eliminate DC fluctuations and high-pass filtered using 8th-order Chebyshev type II, with 20 Hz stop-band frequency to avoid electrical noise interferences. Since filtered signals could still be affected by artefacts, REG signals were visually inspected and sequences with at least 16 consecutive seconds free from interferences were selected within each recording. A total of 53 REG sequences of 16 seconds each free of artefacts were extracted from the 16 volunteer recordings, 29 from apneas and 24 from baseline periods. Fig 2 shows a filtered waveform of a REG signal from an apnea period.

### Analysis of REG pulse wave geometry

**Fig 3:**
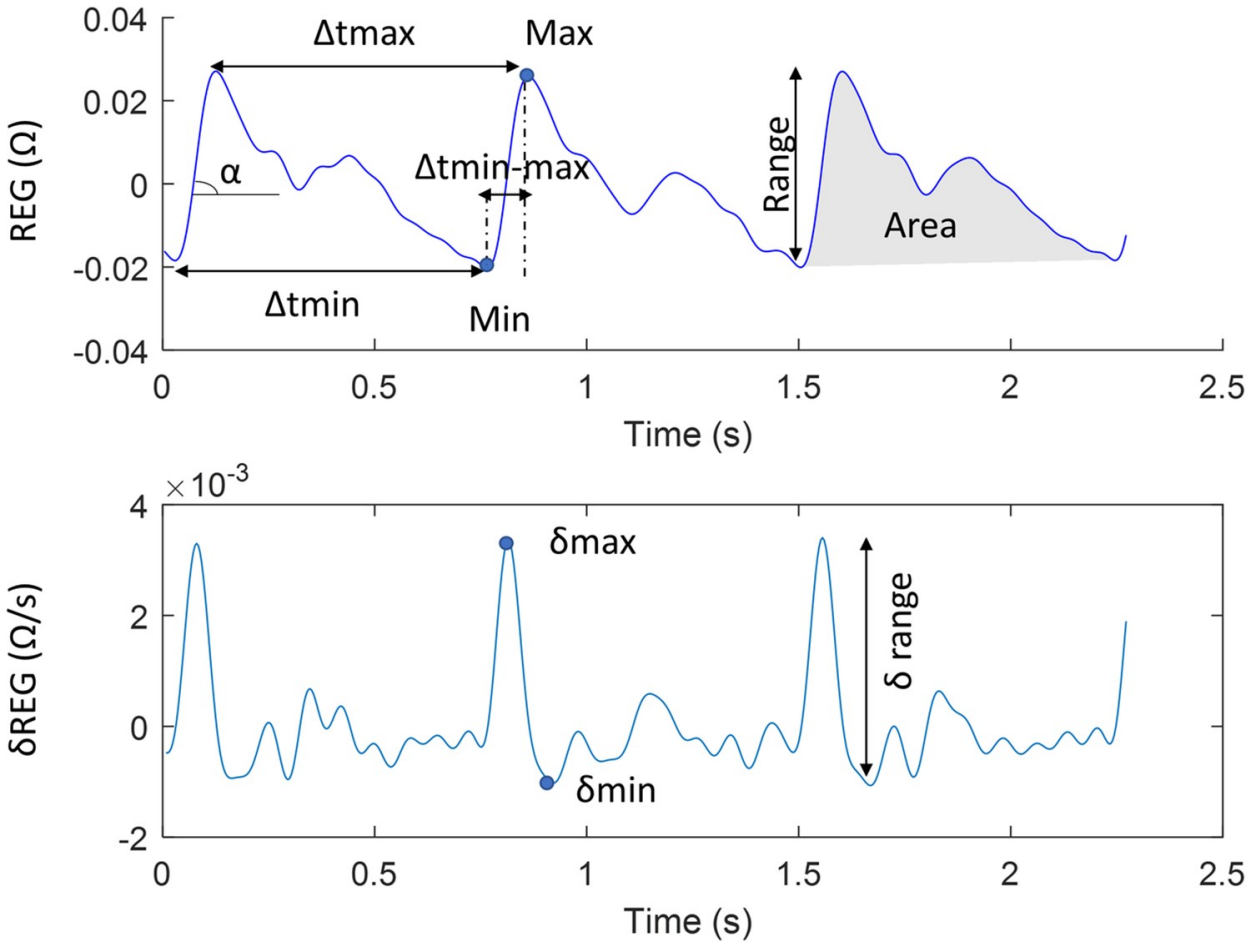
Geometric features extracted from REG pulse waves.

The classical methods used to assess CBF by means of REG signals rely on the analysis of the geometry of the pulse waves. The ability of those methods to distinguish between apnea and baseline signals was studied. For that purpose, minimums and maximums of each pulse wave and their respective derivatives were automatically detected, and the following features were calculated for each signal in the dataset: maximum and minimum pulse amplitudes (Max and Min), amplitude range of the pulse (Range), time between two consecutive maximums (Δtmax), time between two consecutive minimums (Δtmin), time between each minimum and the following maximum (Δtmin-max), the slope of the pulse during this interval (α), the area under the curve (Area), the maximum derivative (δmax) and the range of the derivative (δrange). In each case, to reduce the effect of possible outliers, the median values for all the pulses belonging to each recording was used as a reference. Fig 3 shows the graphical representation of those parameters to be extracted from each sequence.

Results obtained for both apnea and baseline signals were tested for its ability to detect apneas, by means of hypothesis testing, considering significance for p-values<0.05.

### Poincaré plot analysis

Taken’s theorem [23] states that the attractor of a dynamical system can be reconstructed as a state-space representation for a specific time delay (τ) and embedding dimension (m). This attractor, X(t), can therefore be specified as:

X(t)=[x(t)x(t+τ)x(t+2τ)…x(t+(m-1)τ)] (1)

Even though the attractor of a given system might have a high embedding dimension, the analysis of reconstructed attractors on two dimensions has been used extensively to characterize biomedical signals, such as heart rate variability, and has proven to provide relevant information [21,24,25]. In this case, Eq (1) can be simplified as:

X(t)=[x(t)x(t+τ)] (2)

Two-dimensional Poincare plots were constructed from the 16-second REG sequences. Each Poincaré plot is generated with the x-axis representing the REG signal (REG(t)) and the y-axis representing the REG signal after a specified time delay τ (REG(t + τ)), where the length of the series is N and t moves from 1 to N-τ. The time lag τ to be applied to the signal samples to build the Poincare attractor is commonly defined by these criteria:

- 1/4 or 1/5 of the dominant cycle period (T) of the signal [26]
- First local minimum of the auto-mutual information function (AMIF) [27]
- First zero crossing of the autocorrelation function (ACF) [28]
- First value for which the normalized autocorrelation function has a decay of 1/℮ [28].
- First sign change of the second derivative of the autocorrelation function [29]
- 1/10 to 1/20 of the first local minimum of the autocorrelation function [30]

The choice of the time lag τ is critical, since very low values would not allow the attractor to expand, with a majority of points laying on the diagonal line [31], while very large values of τ would cause deformations of the attractor due to the fact that pairs of samples would be uncorrelated [27] [29]. Since no previous work has been done on the analysis of REG attractors, a wide range of τ values was used, from 1 up to the maximum of the above listed criteria, to provide the maximum possible information relating to the dynamics hidden in REG signals.

**Fig 4:**
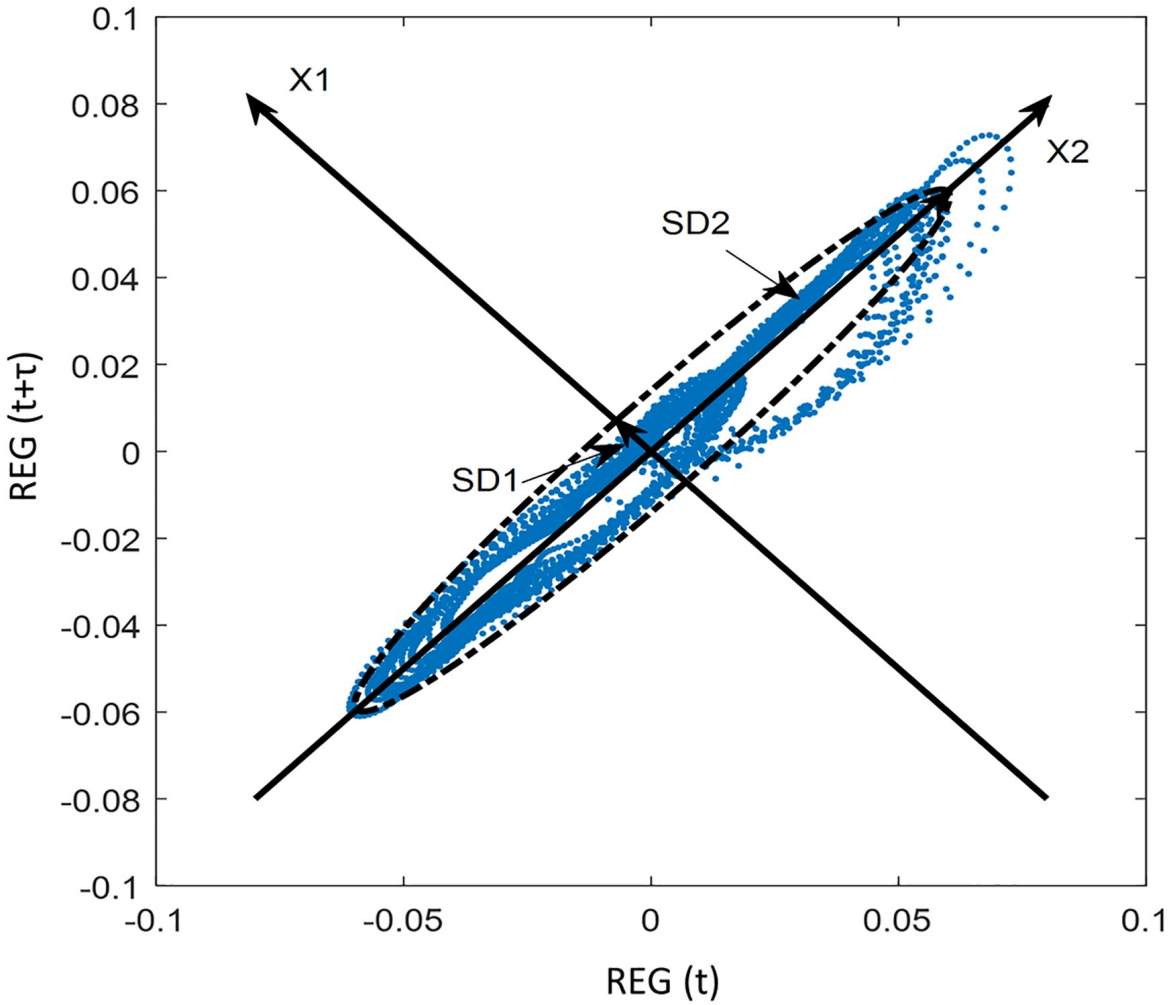
SD1, SD2 and ellipse fitting of a rheoencephalographic (REG) signal.

To generate more quantitative information on the distribution of REG signals in the Poincare plots, several features were extracted from the reconstructed attractor characterization. Two of these features are considered the standard descriptors of Poincaré plots, being named SD1 and SD2. They are obtained by defining a new set of perpendicular axis (x1 and x2 in Fig 4), x2 following the identity line and therefore rotating the axis 45° and fitting an ellipse to the plot as shown in Fig 4. Features SD1 and SD2 are defined as the standard deviation of the distance along the axis x1 and x2 respectively, and lay on the axis of the fitted ellipse having half its size [16,26,32–34]. They are computed following the Eqs (3) and (4), where var is the variance.

SD1=var(REG(t)-REG(t+τ)2) (3)

SD2=var(REG(t)+REG(t+τ)2) (4)

Short term variability is reflected by SD1 while SD2 reflects both, long and short-term variability. Moreover, the area (A) of the ellipse (Eq (5)) has also been considered since it provides a measure of the total variability of the attractor [33].

A=πSD1SD2 (5)

The ratio of SD1/SD2 was also used as a parameter (SDratio) to measure the changes in the scatter patterns. Hayashi et al. [26] proposed this technique as a useful tool for depth of anesthesia assessment by means of Poincaré plots as this ratio reflects the degree of linearity included in the processed signal.

Correlation measures are also proposed to characterize the shape of the Poincaré plots. Eq (6) shows the correlation measure (R) [16], in which E [] is the expected value of the time series and $\overset{-}{REG}$ the average value of the REG(t) time series.

R=E[(REG(t)-REG-)(REG(t+τ)-REG-)]E[(REG(t)-REG-)2]E[(REG(t+τ)-REG-)2] (6)

**Fig 5:**
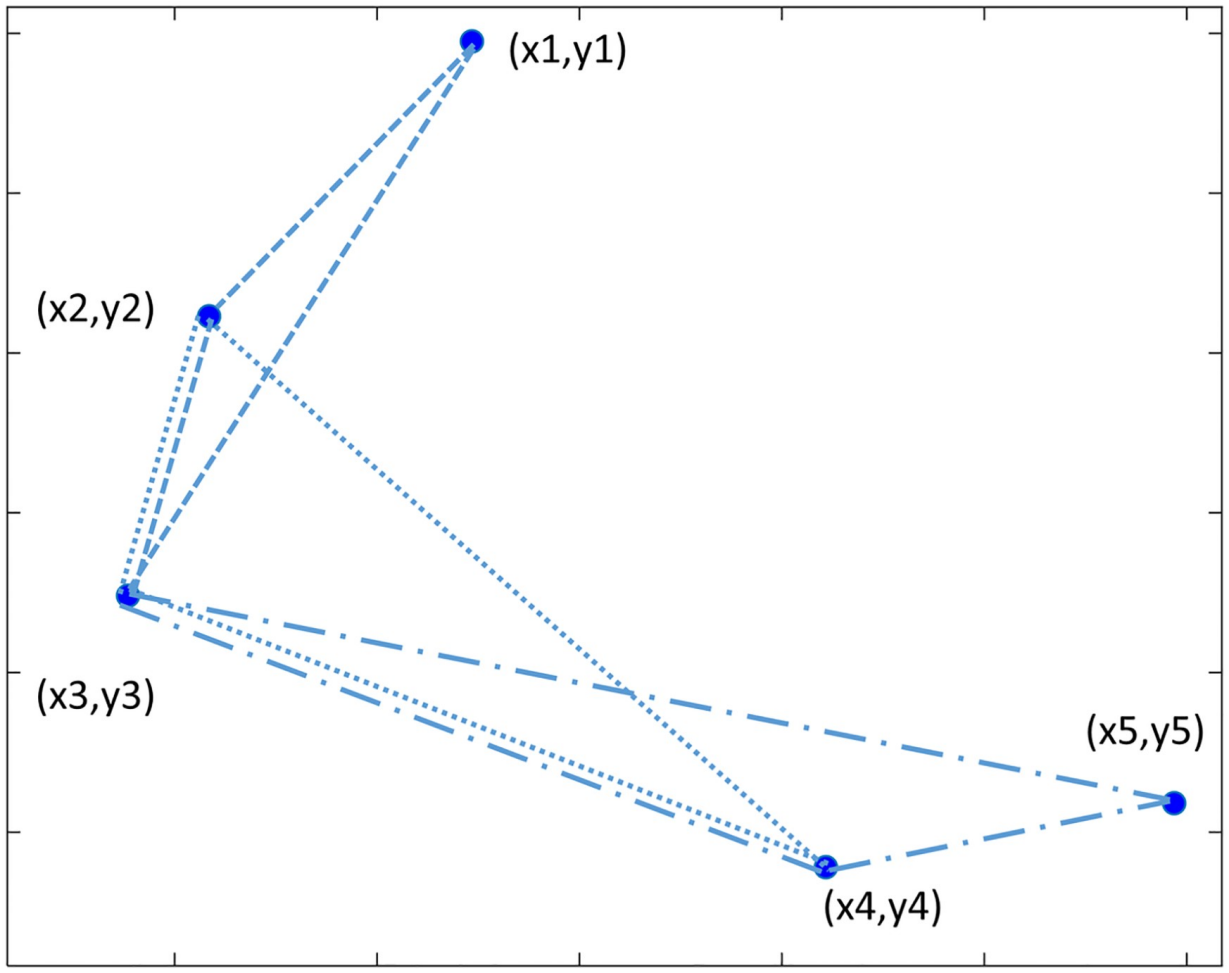
Example of the application of the Complex Correlation Measure (CCM) algorithm in a subset of 5 data points.

Another correlation descriptor considered is the Complex Correlation Measure (CCM) [35]. Its computation identifies all possible sets of three consecutive attractor points of the Poincaré plot and the area of the triangle they define is calculated (Fig 5). In cases where all three points are aligned, the area is considered to be zero. The purpose of analyzing sets of three points in this way is that the descriptor will integrate information from different time lags and instead of reflecting just the overall variance as with SD1 and SD2, it will integrate temporal information as well. CMM is computed as indicated in Eq (7), where N is the number of points in the Poincaré plot, A_n_ is a normalization constant equivalent to the ellipse area (A_n_ = π*SD1*SD2), τ is the time lag of the Poincaré plot and M(i) is the matrix including the coordinates of the three points from each subset whose determinant is the area of the triangle formed by them.

$$\text{CCM}\left(\text{τ}\right)=\frac{1}{{\text{A}}_{\text{n}}\left(\text{N}-2\right)}\sum_{\text{i}=1}^{\text{N}-2}\|\text{M}\left(\text{i}\right)\|$$ (7)

### Statistical analysis and classification procedure

The features extracted from each constructed 2D-Poincaré plot by varying the τ value were SD1, SD2, SDratio, SDarea, R and CCM. A statistical analysis was performed to select τ values that allow 2D-Poincaré plot features to statistically distinguish between signals belonging to apneas and resting periods. Hypothesis testing was applied using student t-test for normal distributions and Mann-Whitney test for non-normal distributions verified by the Kolmogorov–Smirnov test. Significant statistical level was set at p-value<0.05 and Bonferroni correction (p-value<0.025) was applied. The ability of the extracted features to distinguish between apnea and baseline periods were further assessed by means of sensitivity, specificity, area under the curve (AUC) of the receiver operating characteristic and accuracy.

Several algorithms were applied to the features extracted from REG signals, under the scope of classifying them as apneas or baseline recordings. Among all the models available, the present study focuses on logistic regression, naïve Bayes, support vector machines (SVM) and decision trees. While logistic regression aims at estimating the probability of belonging to a defined class, based on the values of one or more predictors, naïve Bayes maximizes the a posteriori probability by applying the Bayes’ theorem, assuming independence among predictors [36]. The logistic regression classifier used in this work is based on a binomial distribution while naïve Bayes was implemented using a Gaussian kernel. Decision trees are based on the classification and regression trees (CART) algorithm [37]. In those algorithms a prediction tree model is built in which each internal node evaluates conditions on predictors, branches represent the output of those evaluations and leaves indicate the class to be assigned to a set of attribute values. Finally, SVM [38] techniques aim to represent attributes in a typically high dimensional space in which data points belonging to different classes are separated, as far apart as possible. When new inputs are represented in the space, their position in the defined hyperplane will define the class they belong to. SVM classifier was implemented with a linear kernel and Sequential Minimal Optimization algorithm as presented in [39].

The four developed classifiers were validated using the leave-one-out strategy. The metrics used to assess the performance of each classifier and compare the results among them were AUC and accuracy.

## Results

### REG pulse waves geometry

Results obtained for the features extracted from the REG pulse waves and their derivatives are summarized in Table 1. Even though some differences can be found between apnea and baseline recordings, none of the selected parameters showed the ability to distinguish between both with statistical significance.

**Table 1:** Results (average for apnea and baseline and obtained p-value).

Parameter	Units	Apnea		Baseline		p-value
		mean	std	mean	std	
**Max**	Ω	0.041	0.014	0.045	0.017	0.356
**Min**	Ω	-0.051	0.017	-0.054	0.018	0.523
**Range**	Ω	0.092	0.028	0.099	0.033	0.376
**Δtmax**	samples	238.7	22.1	254.9	43.3	0.084
**Δtmin**	samples	242.1	23.2	248.6	38.8	0.455
**Δtmin-max**	samples	52.9	27.4	60.6	24.8	0.217
**α**	a.u.	0.002	0.001	0.002	0.001	0.406
**Area**	Ω.s	12.4	4.8	13.5	4.9	0.446
**δmax**	Ω/s	0.006	0.002	0.005	0.002	0.272
**δrange**	Ω/s	0.007	0.002	0.007	0.002	0.145

### Poincaré plot analysis and extracted features

The dominant cycle period of the REG recorded signals, the auto-mutual information and autocorrelation were computed to determine the range of τ values to be used in the reconstruction of the attractors following the criteria previously listed. The average period of REG signals was 0.99 ± 0.12s (mean ± standard deviation), equivalent to 246.9 ± 30.8 samples. No differences were detected between periods of apnea and baseline signals (p-value = 0.345). All values presented for each criterion used for τ calculation are presented in Table 2. On average, the highest τ is provided by the recommendation based on ¼ of the period (61.7 ± 7.7 samples) and therefore the range of τ values used was from 1 to 70 samples (from 0.004 to 0.28 s).

**Table 2:** Tau values (in samples) calculated from the REG signals following the criteria recommended in literature.

	1/4 of the period	1/5 of the period	1st Relati-ve min of AMIF	1st zero of ACF	1/е decay of ACF	2nd deriva-tive of ACF sign change	1/10 of 1st local min ACF	1/20 of 1st local min ACF
Mean	61.7	49.4	34.2	58.4	36.5	19.0	12.0	5.98
Std	7.70	6.16	11.6	14.3	9.73	9.63	3.15	1.57
Min	38.5	30.8	16.0	20.0	14.0	10.0	3.40	1.70
Max	76.9	61.5	70.0	91.0	56.0	49.0	17.5	8.75

Mean, standard deviation (std), min and max values are provided for each criterion.

**Fig 6:**
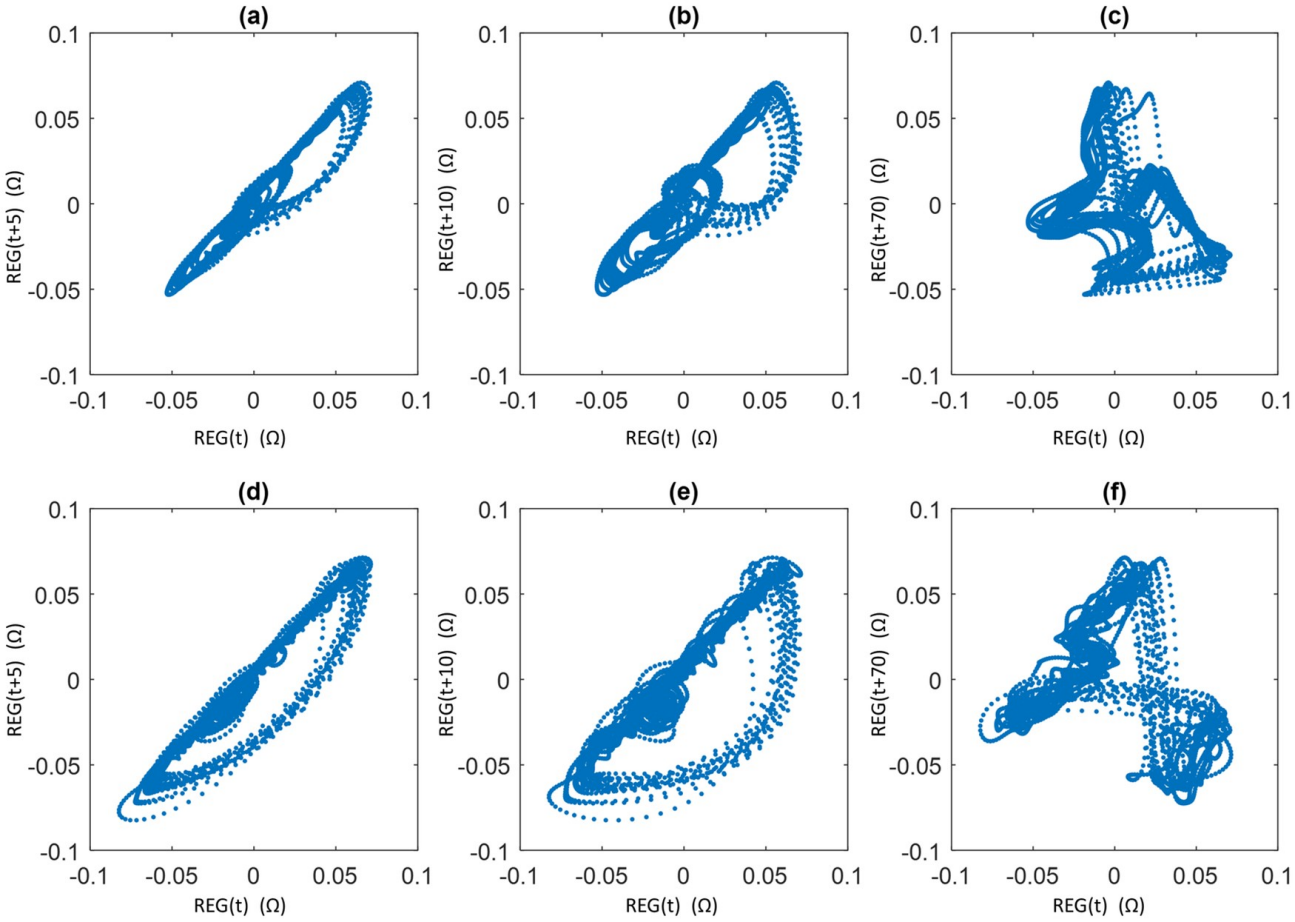
Poincaré plot reconstruction of apnea and baseline signals. Apnea signal (a,b,c) and baseline signal (d,e,f) for different time lags: τ = 5 samples (a,d), τ = 10 samples (b,e) and τ = 70 samples (c and f).

Attractors were reconstructed for every signal and τ value. The two-dimensional Poincaré plots for a baseline and apnea REG signal built for τ = 5, τ = 10 and τ = 70 samples are shown in Fig 6. While low τ values seem to preserve the attractor shape, for τ = 70 samples the attractor looks deformed.

**Fig 7:**
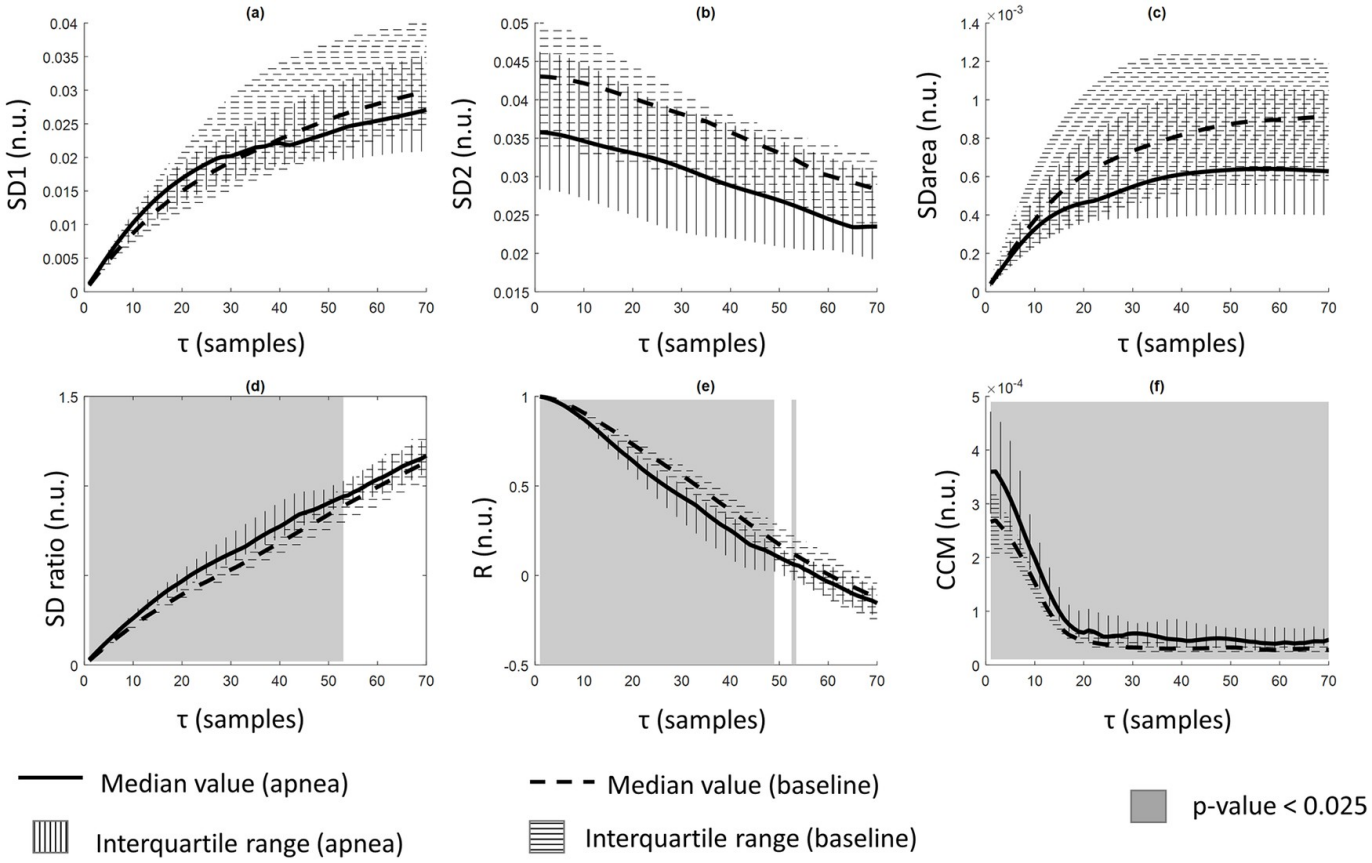
Comparison of the results obtained for apnea and baseline periods (median values, interquartile range and statistical significance) of all tested features in function of the time lag τ. (a) SD1; (b) SD2; (c) SDarea; (d) SDratio; (e) R; (f) CCM. Grey solid color corresponds to statistical significance level of p-value<0.025.

From the reconstructed attractors, the defined Poincaré plot features (SD1, SD2, SDratio, SDarea, CCM and R) have been calculated for every selected segment and every time lag, and their ability to separate apnea from baseline signals has been assessed through hypothesis testing. The evolution of all parameters, as a function of the chosen time lag τ, for both apneas and baseline can be observed in Fig 7.

SD1 shows a curvilinear increase as τ increases (Fig 7a) for both apneas and baseline periods, presenting higher values in apneas for low τ values and reversing this behavior for τ values above 40 samples. The behavior of SD2 is the opposite (Fig 7b), decreasing as τ increases, providing higher values for baseline periods for all tested τ values. None of those features show significant differences between the apnea and baseline groups.

SDarea, which is a composite measure of SD1 and SD2 (Fig 7c), increases with an exponential pattern while τ increases and provides higher values for the baseline group, even though differences are not significant. However, SDratio (Fig 7d) is also a composite feature and shows significant differences between apnea and baseline periods for τ up to 53 samples, resulting in higher values for the apnea group.

The values of parameter R have an opposite behavior when compared to SDratio (Fig 7e), decreasing as τ increases, with the baseline group showing higher values than the apnea group. These difference were significant for all τ lower than 49 and τ = 53. Finally, observing the evolution of the values of the parameter CCM, it can be stated that they are higher in apnea group than baseline group with p-value <0.05 for all range of the analyzed time lags.

Among all tested features, three have shown to be capable of distinguishing between apneas and baseline data for several tau values: SDratio, R and CCM. The features R and SDratio showed statistically significant differences between groups for τ values below 50 samples, showing the lowest p-value for τ equal 2 and 3 samples (p-value = 7.01*10^−5^). CCM showed significant differences between groups for all τ values, presenting its minimum value for τ = 13 samples (p-value = 0.00012).

**Fig 8:**
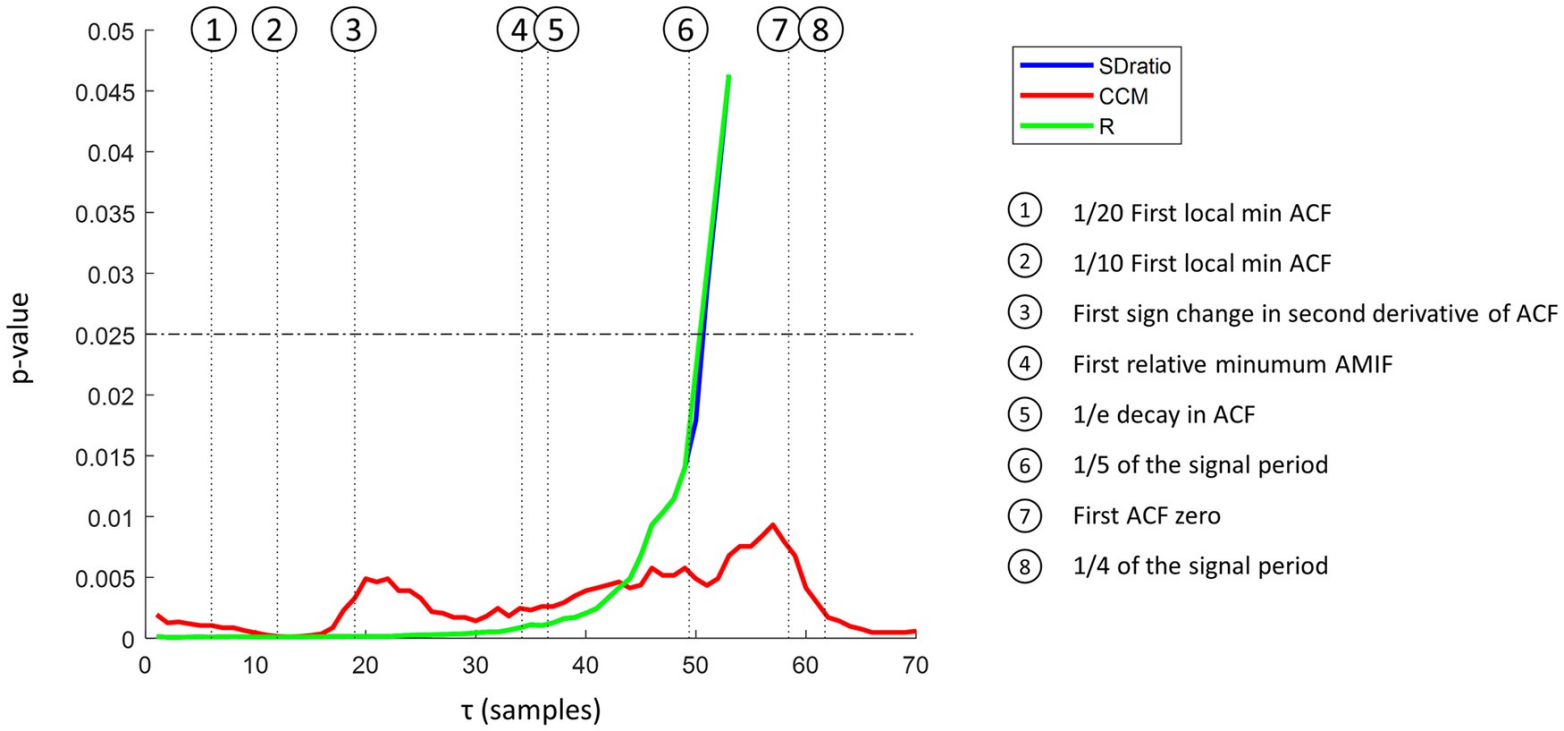
Statistical significance levels (p-value) for SDratio, CCM and R, as a function of the time lag τ when comparing apnea and baseline segments. R and SDratio curves are overlapped for almost all values. The dotted horizontal black line shows significance when applying Bonferroni correction. Vertical lines indicate the different criteria commonly used to determine τ.

Fig 8 illustrates the comparison between the τ values for which the extracted features were significant (Fig 7) with those τ theoretical values presented in Table 2. It can be observed that R and SDratio are statistically different for τ values lower than 50 samples, while CCM is significant for all range with lower values between 10 and 20. It should be noted that for the first six proposed theoretical criteria (1/20 and 1/10 of the first local minimum of the autocorrelation function, the first sign change in its second derivative, the first local minimum of the automutual information, a decay of 1/е of the autocorrelation and 1/5 of the signal dominant period) the three extracted features remain significant, since when using the criterion of 1/5 of the period all p-values are below 0.025.

**Fig 9:**
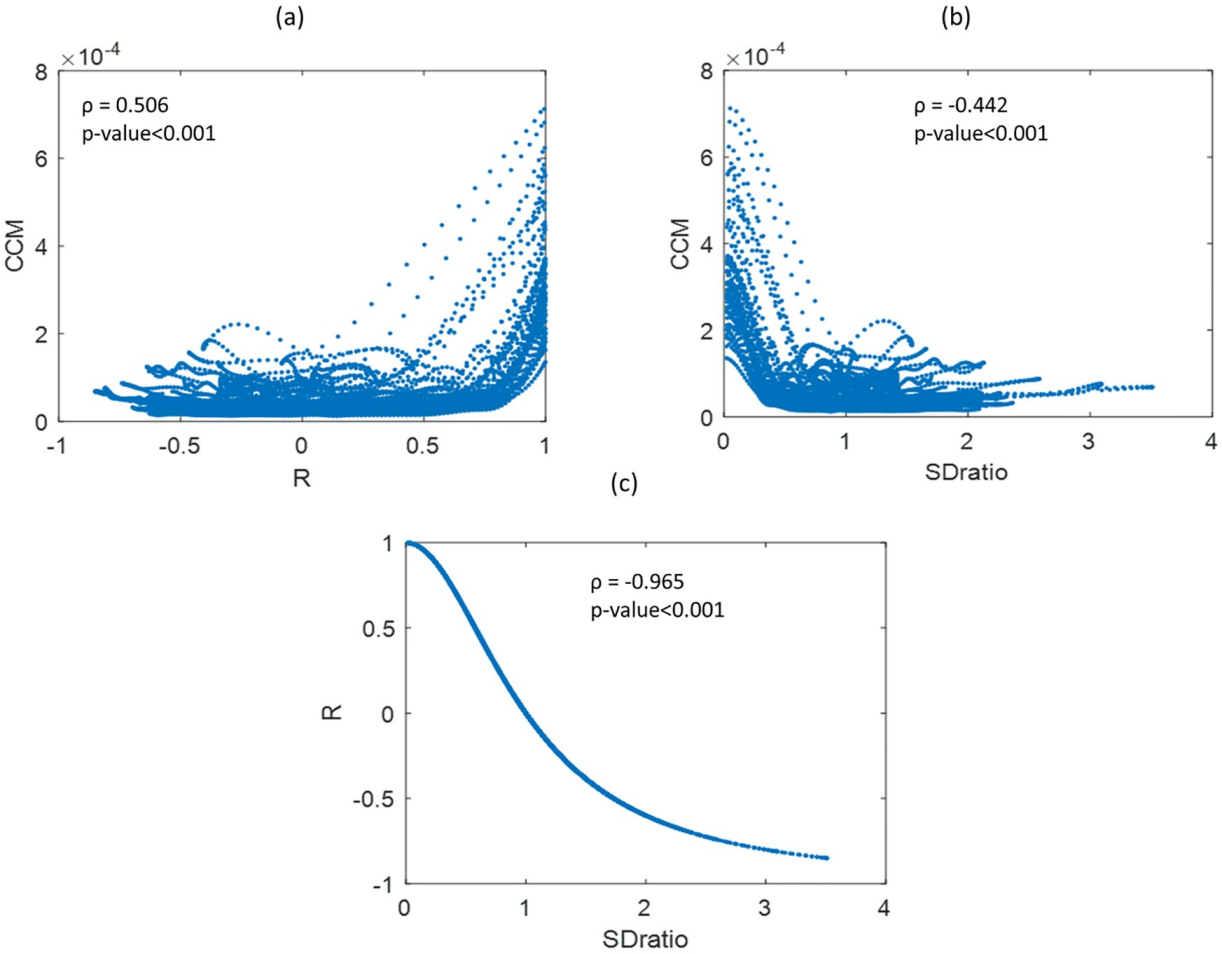
Correlations between R, CCM and SDratio. (a) R and CCM, (b) SDratio and CCM, and (c) CCM and R.

Correlations were performed between features that were statistically significant (Fig 9). Pearson’s correlation was applied to the full dataset including SDratio, CCM and R values from all subjects and all time lags (i.e. resulting in 3710 data points in each case). Since their definitions are quite similar both SDratio and R showed a high correlation (ρ = 0.965, p-value<0.001) although not linear. As can be observed in Fig 9c. CCM showed lower correlations with both SDratio and R.

**Fig 10:**
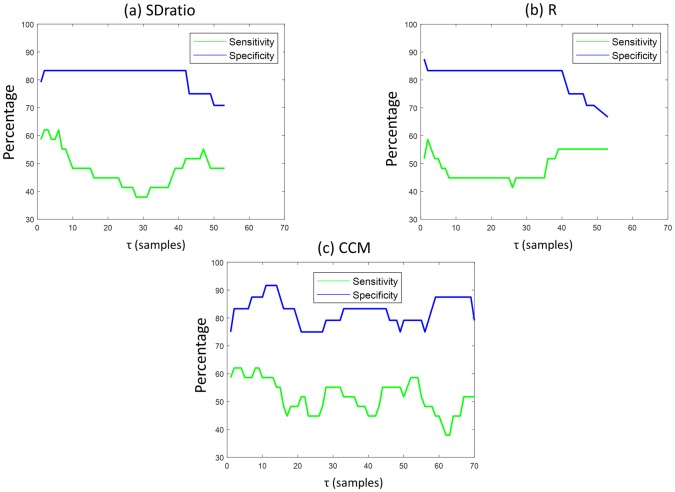
Sensitivity and specificity of apnea and baseline classification as a function of the time lag values (τ). (a) SDratio, (b) R and (c) CCM.

The parameters that showed positive results were analyzed by means of computing their sensitivity, specificity and AUC. Fig 10 shows the sensitivity and specificity for SDratio, R and CCM features in the time lag values for which they showed statistical significant levels. Both SDratio and R provide a high specificity but very low sensitivities, typically below 60% and with higher values for low τ, for which it can be observed that SDratio shows better performance. CCM shows better sensitivity and specificity, showing a peak of 90% specificity for τ between 10 and 20, and maximal sensitivity for τ lower than 10.

For all three, the best performances are detected for very small τ values (lower than 5 samples), lower than the ones proposed in the literature for other physiological signals shown in Table 2, the closest approximation being a time lag corresponding to 1/20 of the first local minimum of the autocorrelation function.

**Fig 11:**
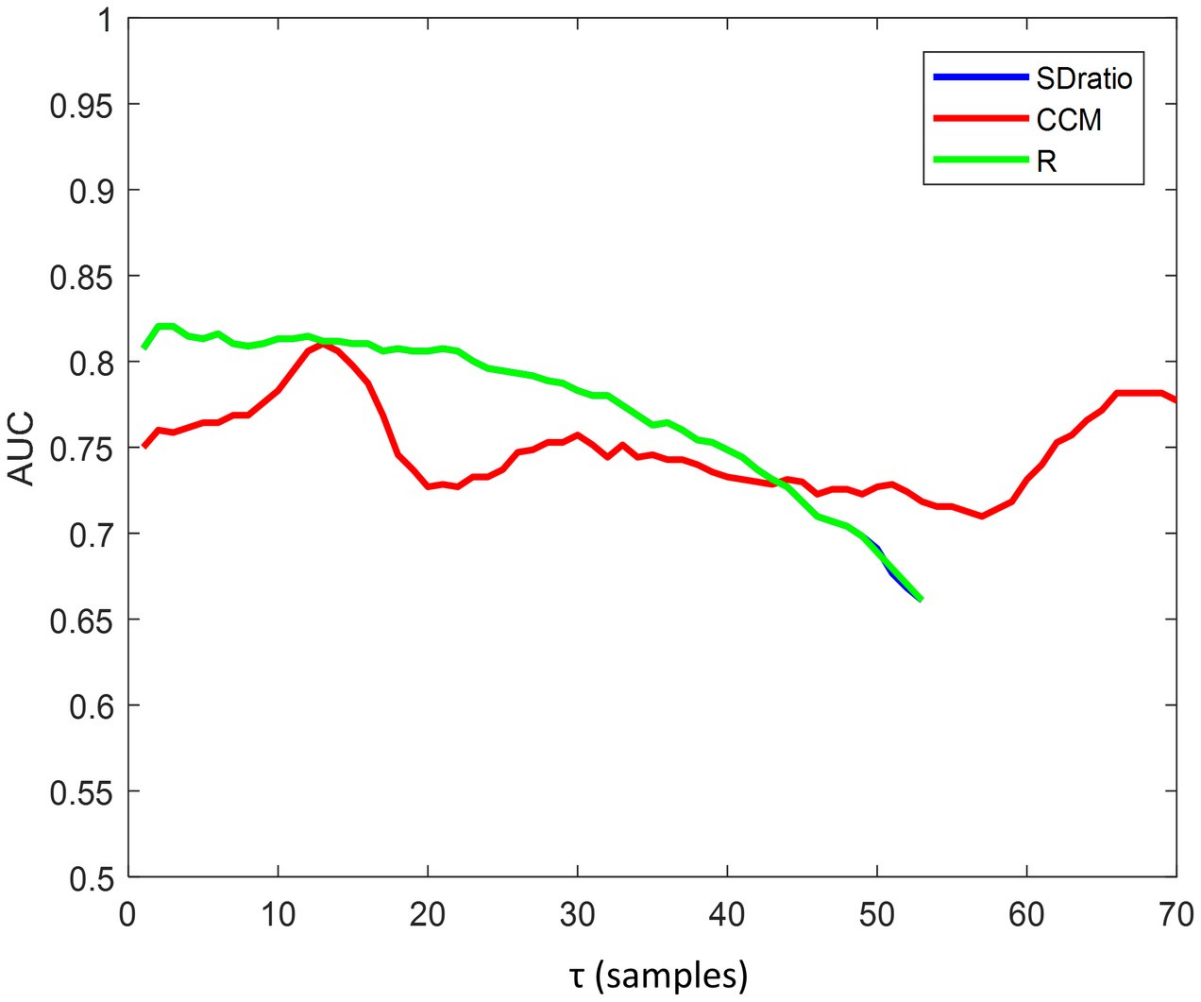
AUC of the receiver operating characteristic for SDratio, CCM and R, as a function of the time lag. AUC for R and SDratio are overlapped.

Fig 11 presents the AUC computed for each parameter and each time lag τ. Both SDratio and R provide the best results, with values higher than 0.8 for low τ values (τ < 5 samples) and decreasing when τ increases. Even though CCM also offers its best AUC values for low time lags, it does not show such a monotonic decreasing behavior and remains significant for all τ. As previously seen in the analysis of the obtained p-values, SDratio (Fig 7d) and R (Fig 7e) provide optimal results for τ values lower than the ones recommended in the literature (Table 2) while the best performance of CCM takes place for τ values consistent with 1/10 of the first local minimum of the autocorrelation function and the first sign change in its second derivative.

### Classification results

**Fig 12:**
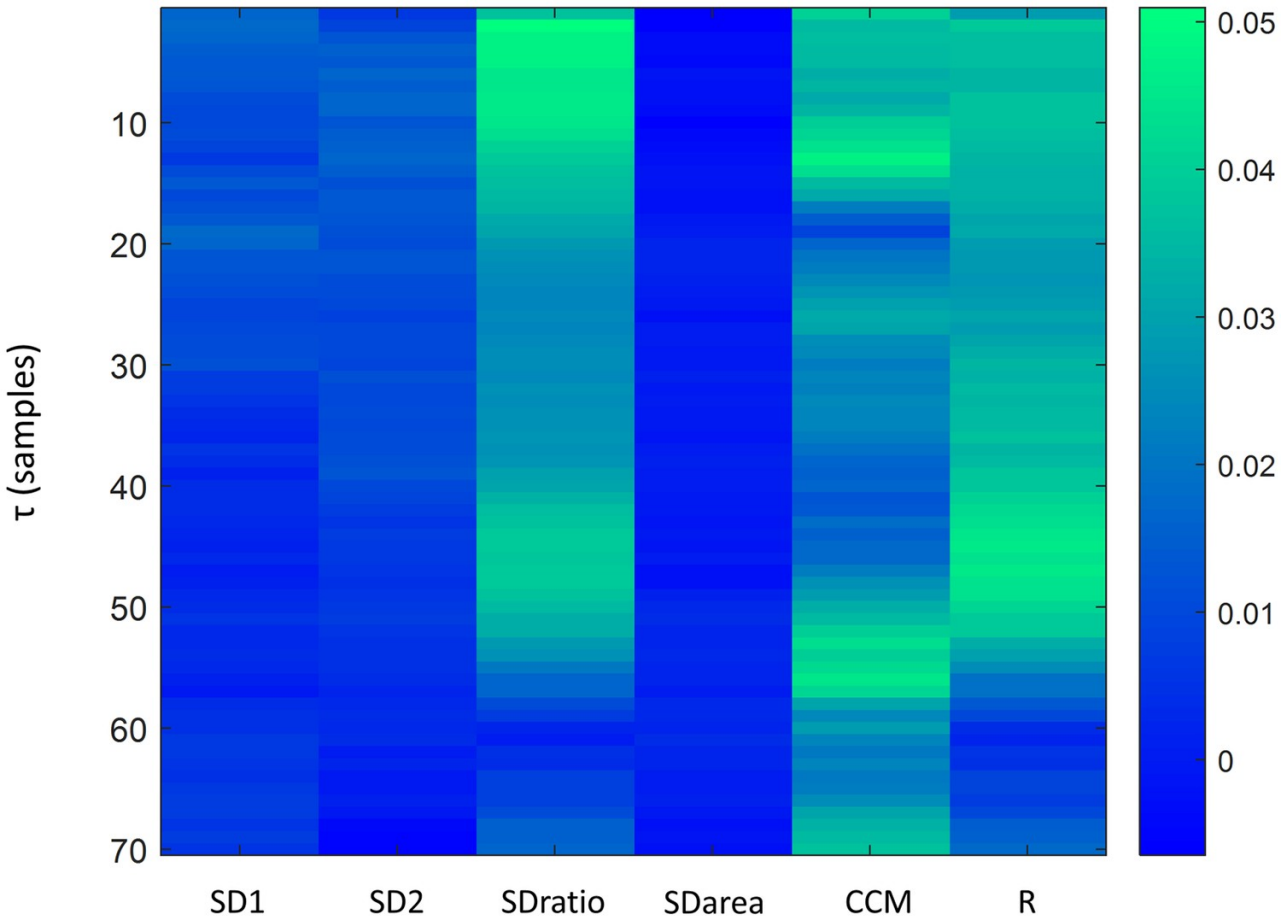
Weights provided by the Relief algorithm for each extracted feature and τ value.

Four different classifiers were tested to assess the possibility of predicting the type of signal (baseline or apnea), considering the information provided by the analysis of Poincaré Plots. A feature selection algorithm, Relief [40], was used to identify the most discriminant features to be considered as inputs to the classifiers. Fig 12 shows the weights provided by the Relief algorithm for each possible input and τ value.

SDratio, R and CCM were the parameters for which the Relief algorithm provided a higher weight and therefore the ones selected as input candidates for the classification model. Each classifier was trained using as inputs all the combinations between the three selected features (SDratio, R and CCM) for each time lag τ. However, since R and SDratio are highly correlated, combinations including both inputs were not considered in the classification process. The results for each of the classifiers with AUC higher than 0.8 and accuracies of at least 80% are summarized in Table 3.

**Table 3:** AUC and accuracy values for the best model of each classifier when classifying apnea and baseline segments.

	Input Variables	Time lag τ (samples)	AUC (mean ± std)	Accuracy (%) (mean ± std)
**Logistic regression**	SDratio	3 ≤ τ ≤ 8	0.814 ± 0.004	81.1 ± 0
	R	4 ≤ τ ≤ 5	0.814 ± 0.001	81.1 ± 0
**Support Vector Machine**	SDratio	3 ≤ τ ≤ 13	0.813 ± 0.003	81.1 ± 0
	R	3 ≤ τ ≤ 7	0.815 ± 0.004	81.1 ± 0
	SDratio and CCM	τ = 3	0.813 ± 0	81.1 ± 0
**Classification Tree**	SDratio	τ = 5	0.927 ± 0	81.1 ± 0
	R	τ = 5	0.927 ± 0	81.1 ± 0
	SDratio and CCM	3 ≤ τ ≤ 5	0.889 ± 0.006	81.1 ± 0
	R and CCM	3 ≤ τ ≤ 5	0.889 ± 0.006	81.1 ± 0

The classification tree showed the best performance, followed by SVM and logistic regression. Naïve Bayes classifier did not provide any results with both accuracy and AUC above the established threshold. The logistic regression classifier provided its best results when inputs were single parameters, either SDratio or R, and for very low τ values, with equal performance for both but a slightly wider τ range for SDratio. Using the SVM method, SDratio and R as inputs optimized the classification strategy with wider τ ranges than logistic regression. A two inputs model based on SDratio and CCM also showed good performance despite being restricted to a single τ value. It is important to note that despite the high correlation between R and SDratio, the AUC and accuracy of the classifiers are affected using SDratio or R, since using R and CCM as inputs for the SVM classifier did not provide positive results.

The classification tree outperformed the other methods, reaching an AUC of 0.927 for τ = 5 when inputs were either R or SDratio, and an AUC of 0.889 when two inputs were used. It can be observed that for this classifier, τ ranges are very narrow and that including the information of the CCM features does not improve the classification results.

## Discussion and conclusions

In this work we have shown that several features extracted from Poincaré plots differ from REG signals extracted from baseline and apnea periods (SDarea, R and CCM). When compared to the performance of the classical parameters based on REG pulse wave geometry, Poincaré related features outperformed the former ones since none of the geometric time domain features showed the ability to detect apneas.

Different time lags (τ) have been tested in Poincaré Plot analysis; lower values optimized the detection of apnea periods both when either a) using single parameters or b) a set of parameters as inputs for classification algorithms. No previous work has been found on the delayed coordinates state-space representation for REG signals therefore results have been compared to the ones provided in publications based on other biological signals, namely heart rate variability. As published by Lerma et al. [21], increasing the time lag should increase SD1, decrease SD2 and therefore increase SDratio. Our findings support these trends for the three descriptors of the reconstructed attractor, showing a curvilinear increase for SD1, a curvilinear decrease for SD2 and a linear increase for their ratio (SDratio). Although SD1 and SD2 did not show any statistically significant differences among apnea and baseline groups, their ratio (SDratio) was able to distinguish between both for τ lower than 53 samples (0.21s). It is noticeable that the SDarea descriptor, which integrates SD1 and SD2 information, evolves in an exponential fashion as τ increases but shows no significant differences between groups. The correlation parameter R, having a definition very close to SDratio and showing a negative correlation close to -1, has the opposite behavior when τ increases, keeping the statistical significance for almost the same τ range.

The CCM feature was also computed for all the reconstructed attractors, showing a monotonic decrease for τ up to 20 samples and a stable value for higher time lags. It demonstrated the ability to distinguish (with statistical significance) between groups for all tested τ values. Karmakar et al. [35] showed that CCM outperformed SD1 and SD2 when applied to heart rate variability signals used to identify arrythmia and congestive heart failures, due to the fact that CCM quantifies the underlying temporal dynamics of the system which are not considered in the definition of the standard Poincaré features. They concluded that a decrease in CCM indicates increased regularity and decreased variability in the signal. In this work, CCM provides better results than SD1 and SD2 when used as a predictor for apneas, but SDratio offers even lower p-values in the hypothesis testing. Therefore, short term (SD1) and long term (SD2) variability are not affected by apneas in a significant manner, but their ratio and CCM value are, which could be interpreted, following Karmakar’s conclusions, as an increased regularity and less variability present in baseline signals.

The significance level of SDratio, R and CCM depends on the time lag used for the attractor reconstruction as shown in Fig 8. The τ values for which the differences among apnea and baseline groups were optimized were compared to the criteria traditionally used for the time lag determination (summarized in Table 2). All those criteria aim at defining a time lag for which the signal samples are still correlated or, in other words, the correlation width [29]. The results from this work show that low τ values provide the best ability to differentiate between apneas and baselines and that for high values, as illustrated in Fig 6 and discussed in other publications [27], the attractor deformation occurs. This suggests that the τ range used (1 to 70 samples) is too wide and that higher τ, instead of further unfolding the attractor, result in a deformation of its underlying structure.

The time lags for which SDratio, and R are optimal are included in the set of τ values recommended in the literature when using 1/20 of the first relative minimum of the correlation as the reference criteria. For CCM the best performance is achieved for τ values closer to 1/10 of the period. Other methods usually applied in literature for other physiological signals propose a set of τ values that would not be suitable for this application. This suggest that those criteria might need to be reviewed for REG signals since the work herein presented indicates that they contain useful clinical information, but it is not available, or maximized, by the same time lags commonly accepted for other applications.

Besides the possibility of using 1/10 or 1/20 of the first minimum of autocorrelation function as the range of τ values to be investigated, the evolution of the CCM parameter over the different time lags suggests that CCM could be a useful reference to determine the maximum τ value for which the system should be tested. The trend of CCM as a function of τ (Fig 7f) clearly shows a first stage in which CCM decreases monotonically until a plateau is reached in both apnea and baseline signals. This inflection point in CCM could be interpreted as the loss of correlation between the signal and its delayed version, presenting CCM as a suitable criterion to identify the range of τ values useful for this application. Furthermore, the inflection point in the CCM trend is consistent with the second derivative sign criteria presented in Table 2 (τ = 19), indicating that optimal range within the 1 to 70 samples interval for all studies parameters is delimited by this upper threshold. Instead of targeting a specific τ value for Poincaré plot analysis, this work suggests that an interval of time lags should be used, which confirms Lerma’s findings [21], and that this interval could be limited by the inflection point observed in the CCM parameter.

The classification tree showed the best performance, followed by logistic regression and SVM. Naïve Bayes classifier did not provide any results with enough accuracy and AUC, suggesting it is not suitable, at least when using a Gaussian kernel, for this application. The best results for each classifier were obtained when using either SDratio or R as single inputs. Even though CCM as a single parameter was proved to be different among both groups, its use as input for the classifiers in combination with SDratio (or R) does not improve the results for any τ value. The behavior of all classifiers was optimal for low τ values (between 3 and 13) with SDratio providing good results for a wider range of τ. As previously discussed, higher τ values do not provide suitable accuracy and AUC values. This can be explained by the deformation suffered in the reconstructed attractors with elevated time lags.

Two limitations deserve special attention: the reduced sample size and the inexistence of previous results to compare against. Regarding the reduced sample size: while available data are large enough for determining relevant features to distinguish between apneas and baselines, the proposed dataset might compromise the performance of classifiers and its comparison. As previously published [41], AUC calculations present high deviations for small sample sizes and therefore comparisons may be inaccurate. Moreover, the set of inputs for the classifiers have always been restricted within this publication to the features computed for a specific time lag because the available data were insufficient to increase the number of attributes to include. For example, features obtained with different time lags in the same classifier.

The second main limitation is related to the inexistence of previous results which does not allow comparison between the presented findings with other research outcomes of REG signals. However, since information related to REG changes has been obtained by means of feature extraction from the Poincaré plot analysis, the findings indicate that this topic deserves a deeper look with an extended and independent database to validate the models herein presented. Furthermore, since REG signals for apneas and baseline periods have shown to present statistical differences in the features extracted, this suggests that CBF changes provoked by apneas are traceable by means of REG recordings.

Bearing in mind the results of this investigation, and considering that other commonly used techniques for CBF monitoring are expensive and/or invasive, the authors consider that the application of nonlinear techniques to REG signals could allow the use of this technology in clinical practice, once it is non-invasive and affordable.
